# Enhanced Blood Supply Through Lower Body Negative Pressure During Slow-Paced, High Load Leg Press Exercise Alters the Response of Muscle AMPK and Circulating Angiogenic Factors

**DOI:** 10.3389/fphys.2020.00781

**Published:** 2020-07-30

**Authors:** Dajana Parganlija, Sebastian Gehlert, Frankyn Herrera, Jörn Rittweger, Wilhelm Bloch, Jochen Zange

**Affiliations:** ^1^Institute of Aerospace Medicine, German Aerospace Center (DLR), Cologne, Germany; ^2^Department of Molecular and Cellular Sports Medicine, Institute of Cardiovascular Research and Sports Medicine, German Sport University, Cologne, Germany; ^3^Department for Biosciences of Sports, Institute of Sport Science, University of Hildesheim, Hildesheim, Germany; ^4^Department of Pediatrics and Adolescent Medicine, University of Cologne, Cologne, Germany

**Keywords:** resistance exercise, robotically controlled leg press, lower body negative pressure, muscle perfusion, physiological responses, AMPK, MMP, endostatin

## Abstract

Lower body negative pressure (LBNP) is an established method of simulating the gravitational effects of orthostasis on the cardiovascular system during space flight or at supine body position on Earth. We hypothesized that LBNP added onto leg press exercise would promote leg muscle perfusion, stimulate oxygen consumption, and modify acute molecular responses. Eighteen subjects performed fifteen slow-paced concentric (4 s) and eccentric contractions (4 s) without or with 40 mmHg LBNP. Force corresponding to 6% of the one-repetition maximum (1-RM) at knee flexion gradually increased to 60% 1-RM within the first half of the range of motion, thereafter remaining constant. AMPK and P-AMPK protein expression was determined in biopsies of vastus lateralis. Venous blood samples were used to measure angiogenic factors. Physiological responses to LBNP included an elevated EMG amplitude, higher heart rate and doubling of the cardiac output compared to control (*p* < 0.001). Muscle total hemoglobin was increased by around 20 μmol/l vs. control (*p* < 0.001), accompanied by decreasing tissue oxygen saturation and elevated oxygen uptake (*p* < 0.05). MMP-2 levels were reduced, and the ratio of P-AMPK to AMPK elevated after exercise with LBNP (*p* < 0.05). MMP-9 similarly increased in both groups, whereas endostatin was only elevated in the control group (*p* < 0.05). Our results indicate facilitated peripheral blood supply and higher oxygen exploitation leading to activation of the energy sensor AMPK and differential regulation of angiogenic factors involved in muscle tissue remodeling and capillary growth. Simulating orthostasis with LBNP might promote beneficial structural adaptations of skeletal muscles during resistance exercise and contribute to future exercise countermeasures achieving increased muscle strength and endurance during space flight.

## Introduction

One of the prevalent issues astronauts face during space flight is the redistribution of fluids toward their upper body, a phenomenon that occurs due to the lack of gravity in space (Hargens and Richardson, 2009; Hargens et al., 2013). As a consequence of this headward fluid shift, the blood supply to the lower limbs is reduced compared to the scenario that humans are naturally adapted to on Earth. Considering an additional difficulty of providing adequate loading, skeletal muscles are presented with a difficult challenge that leads to structural alterations and loss of muscle mass through prolonged exposure to microgravity. This challenge is yet to be fully tackled by countermeasures and represents a particular difficulty when considering long duration space missions, such as traveling to a different planet (Narici and De Boer, 2011; Thornton and Bonato, 2017; Dillon et al., 2018).

Lower body negative pressure (LBNP) is a well-known method of simulating the cardiovascular and physiological effects of gravity and has been used to assess the function of the cardiovascular system before and after space flight (Baisch et al., 2000; Hargens and Richardson, 2009; Hargens et al., 2013). Coupled with exercise, LBNP has shown beneficial effects including the replication of the physiological responses and ground reaction forces of upright gait during supine walking and running on a vertical treadmill, as well as enhanced performance during supine dynamic leg exercise (Eiken, 1988; Boda et al., 2000; Hargens et al., 2013). Combination of LBNP and exercise is a proposed countermeasure against deconditioning in long duration space flight (Murthy et al., 1994). Physiological effects of LBNP such as reduced stroke volume and cardiac output, enhanced heart rate, increased blood flow and elevated muscle tissue hemoglobin have been extensively investigated (Eiken, 1988; Berdeaux et al., 1992; Boda et al., 2000; Zange et al., 2008; Bartels et al., 2011; Blaber et al., 2013; Hargens et al., 2013). Molecular responses to LBNP have so far been investigated, among others, for catecholamines, volume regulating hormones, markers of renal function and blood coagulation (Baily et al., 1991; Maillet et al., 1996; van Helmond et al., 2015; Cvirn et al., 2019; Goswami et al., 2019). Exercise with LBNP has been found to cause an increase in catecholamines and a reduction of lactate levels, as well as to promote phosphocreatine resynthesis leading to improved fatigue resistance (Bonde-Petersen et al., 1984; Eiken, 1987, 1988; Gasiorowska et al., 2005; Zange et al., 2008). Supine exercise on a treadmill with LBNP complemented by further resistive exercise during head-down tilt bed rest was found to promote bone formation and affect the NOS/NO signaling and proteolysis in female skeletal muscle (Salanova et al., 2008; Smith et al., 2008). However, molecular responses to LBNP are not yet fully understood, particularly in combination with resistance exercise, which is the topic of our current work.

Changing body posture alters the gravitational influence on the muscle and is accompanied by altered neuromuscular control. In order to avoid possible interference with the focus of our research, altered blood supply to the working muscle, we developed an electrically driven and robotically controlled leg press within an LBNP-chamber, which can be used for exercise in a supine position (Hargens et al., 2013; Parganlija et al., 2019). This device allows studying the effects of orthostasis without changing the biomechanical properties of exercise, as in case of an actual change of posture. Our current study represents to some extent a continuation of our prior research into the effects of LBNP on leg press exercise, in which we demonstrated enhanced oxygen availability in the working muscle (Parganlija et al., 2019). It is well described that resistance exercise prevents atrophy of unloaded lower limb muscles and can promote their hypertrophy (Tesch et al., 2004). Furthermore, adaptive responses can be enhanced by reduced velocity during strength training (Schuenke et al., 2012). Our focus therefore remains on high load, low velocity contractions as described in our previous research paper. Exercise and oxygen availability in the muscle tissue both influence vital cell functions through molecular mediators such as matrix metalloproteinases (MMPs), endostatin and AMP-activated protein kinase (AMPK), which we investigate in our present study.

MMPs form a large and heterogeneous family of zinc-dependent endopeptidases which essentially degrade proteins of the extracellular matrix by cleaving internal peptide bonds (Lo Presti et al., 2017). They are generally involved in various biological processes including cell migration, growth and differentiation, and tissue remodeling. MMPs are stored in the extracellular matrix of the skeletal muscle tissue and mediate its adaptive responses to exercise, including angiogenesis (Kjaer, 2004; Suhr et al., 2007; Ross et al., 2014). While the remodeling of extracellular matrix through MMPs generally promotes angiogenesis by making way for the expansion of capillaries (Prior et al., 2004), the proteolysis of collagen 18 releases endostatin, which acts as an anti-angiogenic signaling molecule (Page-McCaw et al., 2007). Results of studies investigating the response of endostatin to exercise stimuli vary, possibly depending on the exact type of stimulus and the physical fitness of the study participants. Endostatin levels have been found altered both by endurance and resistance exercise (Suhr et al., 2010; Beijer et al., 2013), although there are also studies that suggest no influence of exercise on endostatin (Rullman et al., 2007).

MMP-2 and MMP-9 are known as gelatinases due to their affinity toward denatured collagen (gelatin). Since they are able to hydrolyze the components of the basal lamina surrounding the myofiber sarcolemma, gelatinases play a particularly important role in muscle growth, development and repair (Nascimento et al., 2015; Lo Presti et al., 2017). High intensity and particularly acute exhausting exercise can lead to muscle damage, the extent of which depends on various factors including physical fitness, type and intensity of exercise, as well as oxygen supply (Lo Presti et al., 2017). As muscle injury impacts the myofiber sarcolemma and the basal lamina, it disrupts anchor sites of satellite cells and consequently triggers their activation and involvement in muscle repair. Satellite cells are additionally activated by the hepatocyte growth factor released from the extracellular matrix by gelatinases (Fu et al., 2015). Both resistance and endurance exercise have been shown to trigger a response from various MMPs, including the gelatinases (Rullman et al., 2007, 2009; Urso et al., 2009; Suhr et al., 2010; Hoier et al., 2012; Ross et al., 2014). Capillary shear stress and/or wall tension associated with increases in muscle blood flow and mechanical stress caused by sarcomere length changes during muscle contraction and subsequent relaxation are apparently closely linked to angiogenesis (Hudlicka et al., 1992; Prior et al., 2004; Kissane and Egginton, 2019). Moreover, increased blood flow seems to be one of the more probable stimuli of angiogenesis in exercise-trained muscles (Brown and Hudlicka, 2003).

AMPK is an abundant, phylogenetically conserved energy-sensing regulator activated by an increase in the cellular AMP content that can occur due to curbed ATP production under glucose deprivation or hypoxia, or through increased energy expenditure, e.g. due to muscle contraction (Winder, 2001; Kahn et al., 2005; Richter and Ruderman, 2009). Different types of exercise induce an isoform-specific increase in activated AMPK that appears to be dependent on the exercise intensity (Fujii et al., 2000; Wojtaszewski et al., 2000; Winder, 2001; Chen et al., 2003). AMPK then adjusts cellular metabolic pathways to the altered energy state in order to prevent high-energy phosphate depletion. In this regard, AMPK triggers energy-producing processes like fatty-acid oxidation and glucose uptake. It also reduces energy-expending pathways not acutely required for cell survival, such as lipid and protein synthesis and pathways involved in cell growth and proliferation. Downstream targets of AMPK are involved both in the regulation of short-term metabolic responses as well as chronic adaptation to exercise (Winder, 2001). AMPK is also involved in other vital cellular processes including mitochondrial biogenesis and angiogenesis as well as the systemic energy expenditure regulated at the level of the hypothalamus (Nagata et al., 2003; Minokoshi et al., 2004; Ouchi et al., 2005; Jäger et al., 2007; Richter and Ruderman, 2009; Klaus et al., 2012). Hypoxia has previously been described as one of the triggers of AMPK activation (Mu et al., 2001; Jørgensen et al., 2006). Furthermore, mechanical unloading as a ground-based model of microgravity has also been shown to impact the phosphorylation of AMPK (Vilchinskaya et al., 2018).

Our present study is an expansion of our previous research that suggested possible stimulation of oxidative metabolism through LBNP (Parganlija et al., 2019) and addresses the physiological and molecular responses to LBNP superimposed on intense, resistive leg press exercise. Based on current scientific knowledge and our own previous findings, we expected to find elevated total hemoglobin levels contributed from the arterial side, accompanied by increased overall oxygen uptake and possibly a lower rise of post exercise lactate levels under LBNP. We hypothesized that increased oxygen availability would promote energy-producing oxidative metabolism, possibly resulting in better maintenance of energy levels and consequently a reduced recruitment of motor units reflected in a lower EMG amplitude increment during exercise. Higher oxygen supply and the stimulation of oxidative metabolism would likely affect the activation of the energy sensor AMPK that adjusts the cellular metabolism depending on energy demands and the presence of hypoxia. Angiogenic factors would expectedly be impacted by several aspects of our experimental setting, as angiogenesis has been linked to exercise, increased blood flow and hypoxia. Our present research provides insights into the, to our knowledge, yet unexplored pathways of molecular responses to LBNP affecting the adaptational mechanisms of skeletal muscle energy metabolism and tissue remodeling. Our findings are relevant for understanding the biology of the orthostatic response as well as for future targeted development of countermeasures for muscle loss in space.

## Materials and Methods

### Study Participants

The study was completed by 18 healthy male subjects with similar profiles of moderate recreational physical activity (for anthropometric data, see Table 1).

**TABLE 1:** Study participants.

Characteristics	Group	
	control (*n* = 9)	LBNP (*n* = 9)
Age (years)	24.4 ± 4.4	22.2 ± 2.9
Body mass (kg)	74.8 ± 8.6	75.9 ± 6.4
Body height (cm)	178.3 ± 8.5	177.3 ± 6.8
1-repetition maximum (N)	2207 ± 398	2325 ± 464

*Values are means ± SEM.*

Eligibility of potential participants was assessed based on their physical fitness and medical history. Furthermore, a medical examination was performed aimed at uncovering any excluding conditions, particularly cardiovascular disease and coagulation defects. Other exclusion criteria were orthostatic intolerance, competitive sportsmanship, smoking, diabetes and keloidosis. The medical examination was comprised of a general blood work, a urine test, a 12-channel resting ECG and an overall checkup by a medical examiner, including a comprehensive medical history. The information on type and frequency of physical activity was gathered in personal interviews. Eligible candidates were instructed to report any changes regarding their medical condition during the study period and were also asked to adhere to certain guidelines, similar to those in our previous research (Parganlija et al., 2019). Study participants were asked not to change the type or intensity of their accustomed physical activity. Furthermore, extensive physical activity (e.g., particularly strenuous sports) was not allowed for up to 2 days before the study appointments. Subjects were also asked to abstain from alcohol for the preceding 24 h and to eat 3 h before their session, after which they should only consume water and refrain from further meals. A protein energy drink (Fresubin, Fresenius Kabi) was consumed 2 h prior to the session to ensure sufficient energy levels and an overall comparable metabolic state. In order to avoid withdrawal effects, no caffeine restriction was imposed, and the subjects were rather asked to maintain their usual intake.

All subjects were fully acquainted with the experimental approach and provided a written informed consent prior to their participation. Approval was obtained from the North Rhine Medical Association’s Ethics Committee (*Ethikkommission der Ärztekammer Nordrhein*, Düsseldorf, approval no. 2013426).

### Study Design

The present study was designed to examine acute effects of leg press exercise under lower body negative pressure (LBNP) compared to ambient pressure (control). The subjects were distributed among the two test groups using a ranking list of their one-repetition maximum (1-RM), determined in a separate appointment prior to the exercise session as published for our previous research (Parganlija et al., 2019). The allocation was accomplished by assigning the strongest subject to the LBNP group, then the second strongest subject to the control group, the third strongest subject again to the LBNP group and so forth until the test groups were complete. Physical attributes of the subjects were comparable among the so formed test groups (Table 1).

**FIGURE 1:**
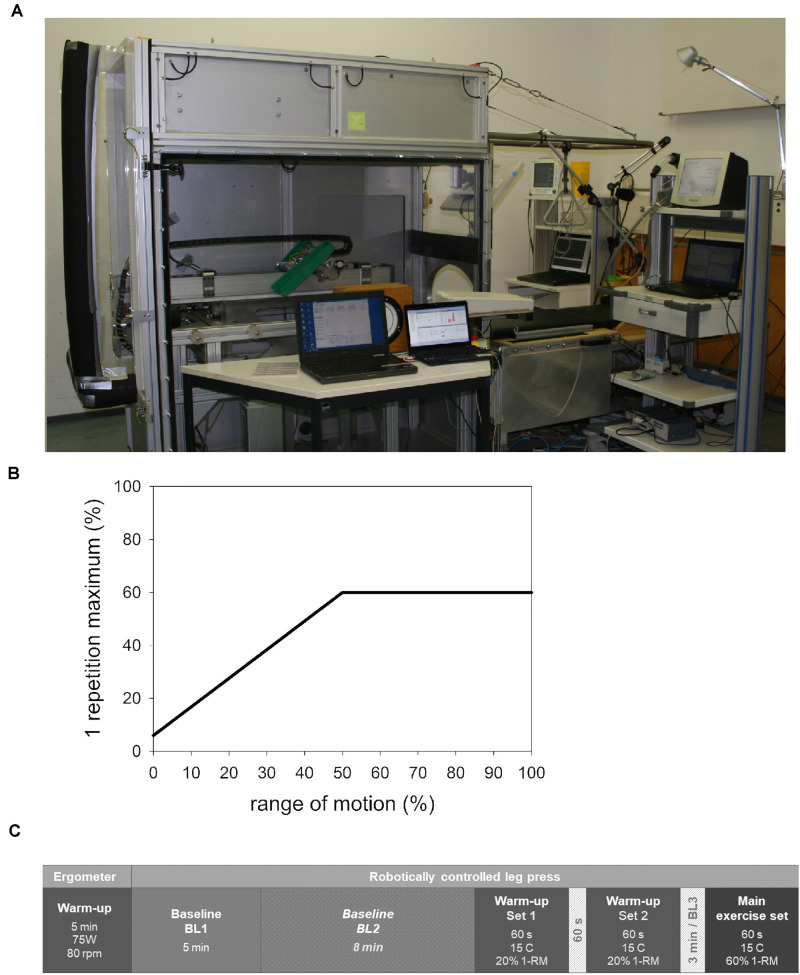
Robotically controlled leg press (RCL) within a lower body negative pressure (LBNP) chamber at the Institute of Aerospace Medicine, German Aerospace Center, Cologne **(A)**. The force-distance profile **(B)** featured a linearly increasing force starting with 6% of the individual 1-RM (i.e., 10% of the target force) at knee flexion and reaching full target force (60% 1-RM) at midpoint of the individual range of motion. From there on, the force remained constant into the terminal point of extension. The described distribution of forces during concentric knee extension was mirrored in the subsequent eccentric work phase. The study sessions consisted of a general warm-up followed by baseline data collection, an exercise-specific warm-up, and the main exercise set **(C)**. The sequence of events in the control and LBNP sessions was equivalent, with the exception of baseline 2 (BL2). This particular baseline was conducted under LBNP and was therefore only part of the LBNP sessions. BL, baseline; C, contraction; 1-RM, 1-repetition maximum.

Both study groups performed the exercise on the robotically controlled leg press with an LBNP chamber (RCL, Figure 1A) developed at the German Aerospace Center (DLR Cologne, Germany) in cooperation with the companies Sensodrive G.m.b.H (Weßling, Germany) and S.E.A Datentechnik GmbH (Troisdorf, Germany). The RCL contains a linear drive controlled by an electromotor that enables safe exercise not requiring energy storage in weights or springs as well as fully customizable force-distance profiles. A detailed description of the RCL is available in the publication on our previous study on this device (Parganlija et al., 2019). In summary, the leg press is located within an LBNP-chamber sealed through a neoprene skirt worn by the subjects, with the seal placed around their hips, in order to maintain stable pressure within the chamber. For the purposes of comparability of the two experimental conditions (control and LBNP) and to avoid any possible effects of omitting the neoprene skirt on the exercise performance, the control group also wore it during their study sessions. Subjects rest their upper body on a backrest outside of the LBNP-chamber, elevated at a 30° angle. Subjects’ feet are placed on pedals attached to the linear drive, with the height adjusted to the length of their lower extremities. The individual range of motion was set so that the knee angle varied between 80° and 125°, allowing almost horizontally directed forces during exercise. The rotational axes of the pedals were located under the tibiotalar joint to avoid loading of the plantar- and dorsi-flexor muscles. A screen attached to the front of the LBNP-chamber provided visual feedback to the subjects concerning the actual and the target leg position, the difference in forces measured at subjects’ feet and the rotation angle of each foot. Subjects were instructed to maintain a neutral foot angle and the comparably low exercise velocity of 8 s per repetition (4 s in each direction within the individual range of motion). Maintaining the target velocity was additionally supported by a metronome and vocal instructions. A contraction cycle started at knee flexion with 10% of the target force (Figure 1B). The force linearly increased during the first half of concentric knee extension, reaching the target force midway through the individual range of motion. From there on, the subjects performed the exercise with the full target force. The force-distance profile during the subsequent eccentric work phase mirrored that of the concentric phase. The LBNP level was 40 mmHg (± 1 mmHg), starting and ending with a ramp of 4 mmHg/s. Considering the risk of syncope under LBNP, blood pressure and heart rate were monitored by an independent physician.

#### Exercise Sessions

The exercise protocol (Figure 1C) corresponds to our previously published research (Parganlija et al., 2019). Exercise sessions started with a general warm-up on the bicycle ergometer (5 min, 75 W, 80 rpm), followed by a 5 min baseline data collection phase on the RCL (BL1). In case of the LBNP exercise sessions, this initial baseline was followed by an additional 8 min baseline phase under 40 mmHg LBNP (BL2), allowing the cardiovascular system to reach a steady state after the volume shift caused by LBNP. The subjects then proceeded with an exercise-specific warm-up comprised of two sets of fifteen contractions with 20% 1-RM. The two sets of warm-up were separated by a 1 min pause. The warm-up was followed by a 3 min pause, of which the last minute provided data for BL3. The subsequent main exercise set was comprised of fifteen contractions (C1-C15) with a target force corresponding to 60% of the 1-RM. Net force and foot position were continuously recorded together with the electromyogram at a sampling rate of 1500 Hz. The obtained data were used to analyze following parameters: measured test force (N) determined as the average force over 2 s around the turning point of extension, mean force (N) averaged over the entire 8 s contraction cycle, the range of motion in one direction (mm), concentric and eccentric velocity (mm/s) as well as concentric and eccentric power (W, power = force × velocity × 0.001). Directly following the completion of the main exercise set, the subjects provided a rating of perceived exertion (RPE) based on Borg’s Scale with a range of 6–20 (Borg, 1998). The subjects were then immediately transported on a stretcher across the short distance to a directly adjacent biopsy-room.

### Electromyography

Muscle activity was measured using the Noraxon Myosystem 1400 electromyograph (EMG; Velamed, Germany). Pairs of electrodes were placed over the vastus lateralis muscle of both legs. As near infrared spectroscopy was also performed on the right leg, the EMG electrodes were positioned distally from the PortaMon device. EMG data were collected at a sampling rate of 1500 Hz. For the concentric phase of each of the 15 contraction intervals, the root mean square of amplitudes was calculated and averaged for both legs (Parganlija et al., 2019). EMG amplitudes are given as percentages of the first contraction.

### Cardiovascular Parameters

Continuous measurement of blood pressure was conducted using a plethysmographic device (Finometer MIDI, Finapres Medical Systems, The Netherlands). The finger cuff was placed on the fourth finger of the right hand. In addition to the blood pressure changes, we report the following parameters derived from the blood pressure data on a beat-by-beat basis using the BeatScope software (ADInstruments, Australia): heart rate (HR, 1/min), stroke volume (SV, ml), cardiac output (CO, l/min) and total peripheral resistance (TPR, dyn × s × cm^–5^). In case of BL1, mean values were calculated across its entire period. For BL2 and BL3, mean values were determined during the last 60 s preceding the respective following phases (Parganlija et al., 2019).

### Muscle Tissue Hemoglobin

Near infrared spectroscopy (NIRS) was used to determine the concentration of oxygenated and deoxygenated hemoglobin (O_2_Hb and HHb, in μmol/l tissue). The telemetric PortaMon device (Artinis Medical Systems BV, Elst, The Netherlands) was positioned over the belly of the vastus lateralis muscle of the right leg. The device was tightly fixed with black elastic kinesiology tape in order to reduce artifacts stemming from motion and outer light sources. The signal was received continuously at a rate of 10 Hz, and the device automatically calculated the total hemoglobin content (tHb = O_2_Hb + HHb, μmol/l) and the tissue oxygen saturation index (TSI = 100 × O_2_Hb/tHb, %). Mean values of the baseline phases were calculated as described above for the cardiovascular parameters. A pattern of recurring minima during the high load phases and maxima during the low load phases of the contraction intervals was observed, as described in our previous research (Parganlija et al., 2019). This pattern reflected the blood flow and variation of hemoglobin oxygenation more precisely than the corresponding mean values. Therefore, we determined a minimum and a maximum value per contraction interval (C1–C15) for each NIRS parameter. As values mostly stabilized by C3, statistical analysis was conducted for C3–C15.

### Respiratory Oxygen Uptake

From the start of BL1 until the end of the main exercise set, respiratory oxygen uptake (V’O2, l/min) was continuously measured in a breath-by-breath mode using the Innocor spirometer (Innovision, Denmark). Data obtained during the last 100 s of the main exercise set were used to determine its mean value, as V’O2 had by then stabilized and almost reached a steady state. ΔVO’2 (l/min), an indicator of the metabolic costs of exercise, was finally calculated by subtracting the mean VO’2 during BL1 from the average value determined for the main exercise set (Parganlija et al., 2019).

### Lactate and Protein Measurements in Blood Samples

Venous blood samples were obtained through a short catheter 30 min before the warm-up on the bicycle ergometer and 10, 30, 60, and 120 min after exercise. Samples for the lactate measurement were collected into sodium-fluoride (NaF) monovettes, and the samples for the protein measurements (endostatin, MMP-2 and MMP-9) were drawn into serum monovettes (Sarstedt, Nümbrecht, Germany). As the target time frame for possible effects on lactate was estimated to not span beyond 30 min, no further samples were collected for the lactate measurement beyond this time point. Blood samples intended for the lactate measurement were immediately placed on ice, and the serum samples were kept at room temperature. Both types of samples were centrifuged within 15 min after their collection (Heraeus Multifuge 1S-R, Thermo Scientific, Waltham, MA, United States), aliquoted and stored at an appropriate temperature (−20°C or −80°C) before analysis.

Venous lactate levels (mmol/l) were determined with the Cobas Integra Lactate Gen.2 enzyme color test (Roche Diagnostics GmbH, Mannheim, Germany). Serum levels of endostatin (ng/ml), total MMP-2 (ng/ml) and MMP-9 (92 kDa Pro-MMP-9 and 82 kDa active MMP-9; ng/ml) were detected in doublets using enzyme-linked immunosorbent assay kits (ELISA; R&D Systems, Wiesbaden, Germany). Measurements were conducted according to the instructions of the respective manufacturer.

### Protein Expression in Muscle Biopsies

Muscle biopsies were obtained from the vastus lateralis using a biopsy rongeur (Zepf, Dürbheim, Germany) or an AceCut biopsy needle (11G, 75 mm). The baseline biopsies were collected 2 weeks prior to the exercise sessions. Further biopsies were obtained 10, 30, and 60 min following the main exercise set. The biopsies were immediately carefully rinsed with saline to remove any traces of blood, frozen in liquid nitrogen and stored at −80°C. Samples were prepared for Western Blot by using a homogenizer (Mikro-Dismembrator, Braun) at maximal speed for 20 s. Homogenized samples were immediately transferred into a lysis buffer mix for protein extraction, containing 10 × Cell Lysis Buffer (New England Biolabs), Phosphatase Inhibitor Cocktail (Thermo Fisher Scientific) and a phenylmethylsulfonyl fluoride (PMSF) and isopropanol solution. Samples were centrifuged at 4°C and 8000 rpm for 20 min, and the amount of protein in the supernatant was determined using the DC Protein Assay (Bio-Rad).

Proteins (15 μg pro lane, in Laemmli Sample Buffer) were separated by SDS-PAGE on Criterion XT Bis-Tris 4–12% gels in XT MOPS Running Buffer and transferred by semi-dry blotting onto PVDF membranes using the Trans-Blot Turbo Transfer System (Bio-Rad). The PVDF membranes were blocked for 60 min in TBST containing 5% BSA in case of AMPK-detection, and 5% milk powder for the detection of P-AMPK or alpha-actin. After rinsing with TBST, the membranes were incubated with one of the following primary antibodies: an AMPK monoclonal antibody (1:500, Cell Signaling Technology 2793S), a phospho-AMPK (threonine 172) polyclonal antibody (1:400, Cell Signaling Technology 2531S) or a polyclonal alpha-actin antibody (1:5000, Sigma-Aldrich A2066-.2ML). After further washing with TBST, the membranes were accordingly incubated with a secondary mouse or rabbit antibody (1:8000, Cell Signaling Technology 7076S or 7074S, respectively). Horseradish peroxidase activity on the secondary antibodies was detected with the SuperSignal West Dura Extended Duration Substrate (Thermo Fischer Scientific). Protein bands were visualized using the ChemiDoc XRS + (Bio-Rad) and quantified with the accompanying Image Lab 5.2 software.

### Statistical Tests

Linear mixed effects models and *t*-test were used to determine the significant effects of the test conditions (control and LBNP) and time point, where appropriate. SPSS (IBM SPSS Statistics Version 21) was used for statistical analysis. In case of data presented as percentages, calculation was performed using the individual data of each subject to determine the percentage of the respective baseline. Reported changes are an average of the so determined individual percentages. The individual percentages were used for statistical analysis. Results above the threshold of significance obtained by LME are reported as follows: F (degrees of freedom numerator/degrees of freedom denominator) *f*-value, *p*-value. The *p*-values above the threshold of significance obtained by *t*-test are reported together with Cohen’s *d*, under the following assumptions concerning the effect size: *d* ≤ 0.2, small effect; 0.2 < *d* < 0.8, medium effect; *d* ≥ 0.8, large effect. Data are presented as mean ± standard error (SEM) or standard deviation (SD), as indicated in the accompanying text. Box and whisker plots with SD are available as Supplementary Material. Effects with *p* < 0.05 were rated as statistically significant.

## Results

### Exercise Protocol Implementation

Test and mean force, work distance, concentric and eccentric velocity, as well as corresponding power during resistive leg press exercise were not significantly altered by superimposed LBNP, indicating equivalent exercise performance under control and LBNP conditions (Table 2).

**TABLE 2:** Exercise parameters.

Parameter	Exercise sessions		*p*-value
	control (*n* = 9)	LBNP (*n* = 9)	
Test force (N)	1171 ± 212	1229 ± 234	0.589
Mean force (N)	808 ± 147	847 ± 139	0.573
Range of motion (mm)	214 ± 33	199 ± 43	0.421
Concentric velocity (mm/s)	53 ± 8	50 ± 11	0.421
Eccentric velocity (mm/s)	−54 ± 8	−50 ± 11	0.355
Concentric power (W)	50 ± 12	49 ± 13	0.833
Eccentric power (W)	−46 ± 11	−45 ± 12	0.890

*Values are means ± SEM. Test force was determined based on the values during the high load phase of each contraction cycle and averaged across the main exercise set, during which the target force was set to 60% of the subjects’ 1-RM. Mean force values were determined based on the entire contraction cycle and also averaged across the main exercise set.*

Majority of subjects completed their 15 repetitions as defined in the training protocol. Three subjects of the LBNP-group were unable to fully complete their fifteenth repetition, while indicating a rating of perceived exertion (RPE) of 20 (maximum value), 17 and 15 based on Borg’s scale (Borg, 1998). Subsequent to the main exercise set, the overall RPE was 17.0 ± 2.1 (mean ± SD) for control and 17.9 ± 1.5 for the exercise under LBNP, displaying no significant difference between the two test conditions (*p* = 0.324). The exercise strongly challenged the endurance and strength of all subjects, as intended by the designed training protocol. As the achieved exposure was sufficient to uncover possible effects of LBNP and considering the otherwise equivalent performance between the test groups, the described minor deviation from the test protocol in the three subjects of the LBNP-group was not considered critical as to omit their results from the overall findings.

### EMG Amplitude Increment

**FIGURE 2:**
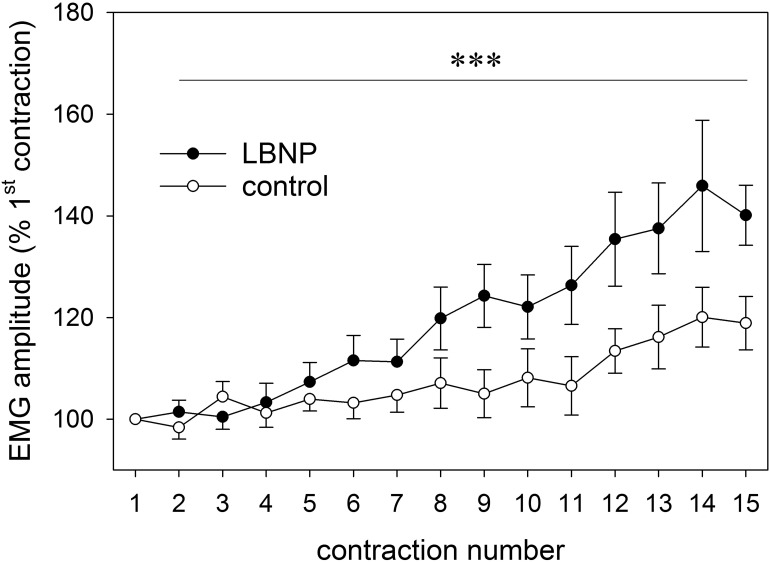
EMG amplitude of the vastus lateralis of both legs (as percent of first contraction, mean ± SEM) determined across the full range of concentric contractions (1–15) on the RCL, under ambient pressure (control, ∘; *n* = 9) or with LNBP (∙, *n* = 9). ^∗∗∗^*p* < 0.001, LBNP vs. control.

Relative increase in the EMG amplitude of the vastus lateralis muscle gradually became elevated over the course of the main exercise set. Under LBNP, this occurred as of the sixth contraction, whereas under ambient pressure a marked increase only took place later during the exercise set, starting at the twelfth contraction. The EMG amplitude increase not only occurred earlier, but was also overall higher with superimposed LBNP (*F* (1/179.5) 30.4, *p* < 0.001, Figure 2).

### Cardiovascular Responses

**FIGURE 3:**
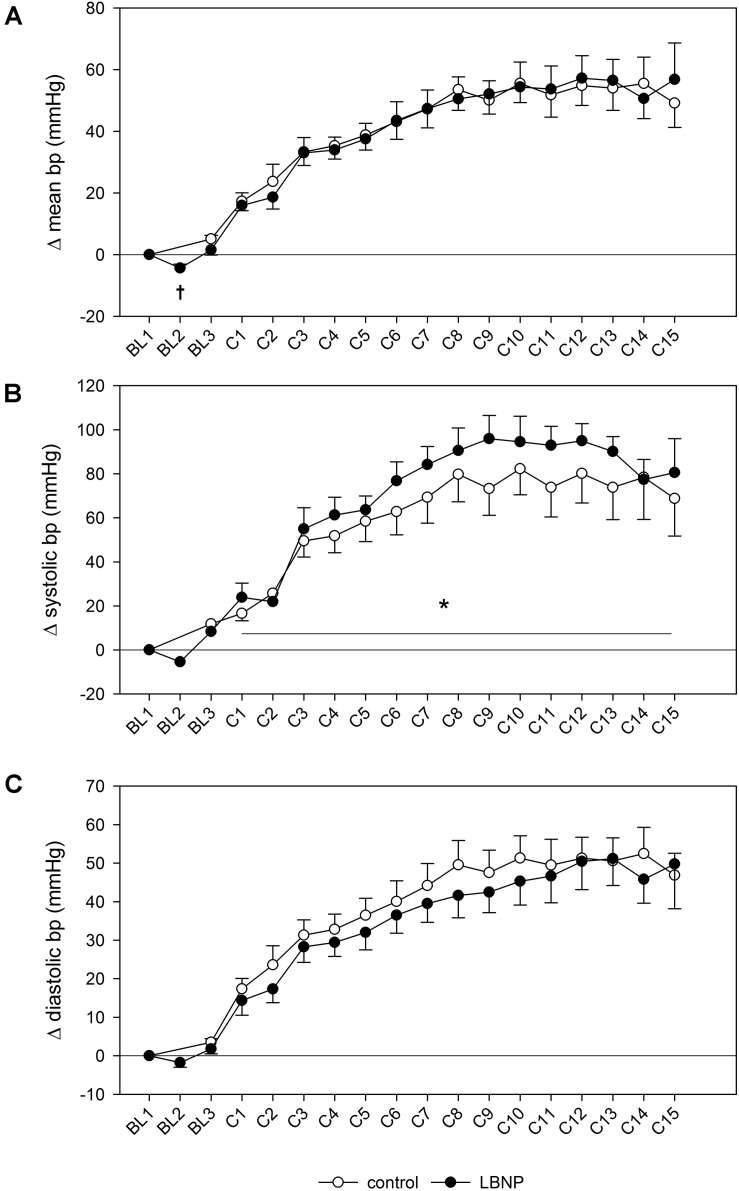
Changes in mean blood pressure **(A)**, as well as systolic **(B)** and diastolic blood pressure **(C)** measured by finger plethysmography during a single set of resistive leg press exercise (contractions C1–C15) under ambient pressure (control, ∘; *n* = 9) or with LNBP (∙, *n* = 9), relative to onset baseline values (BL1, Δ). Values are means ± SEM. BL2, LBNP onset; BL3, baseline directly preceding the main exercise set. Significant effects are marked as follows: † LBNP vs. baseline (*p* < 0.05), *LBNP vs. control (*p* < 0.05).

**FIGURE 4:**
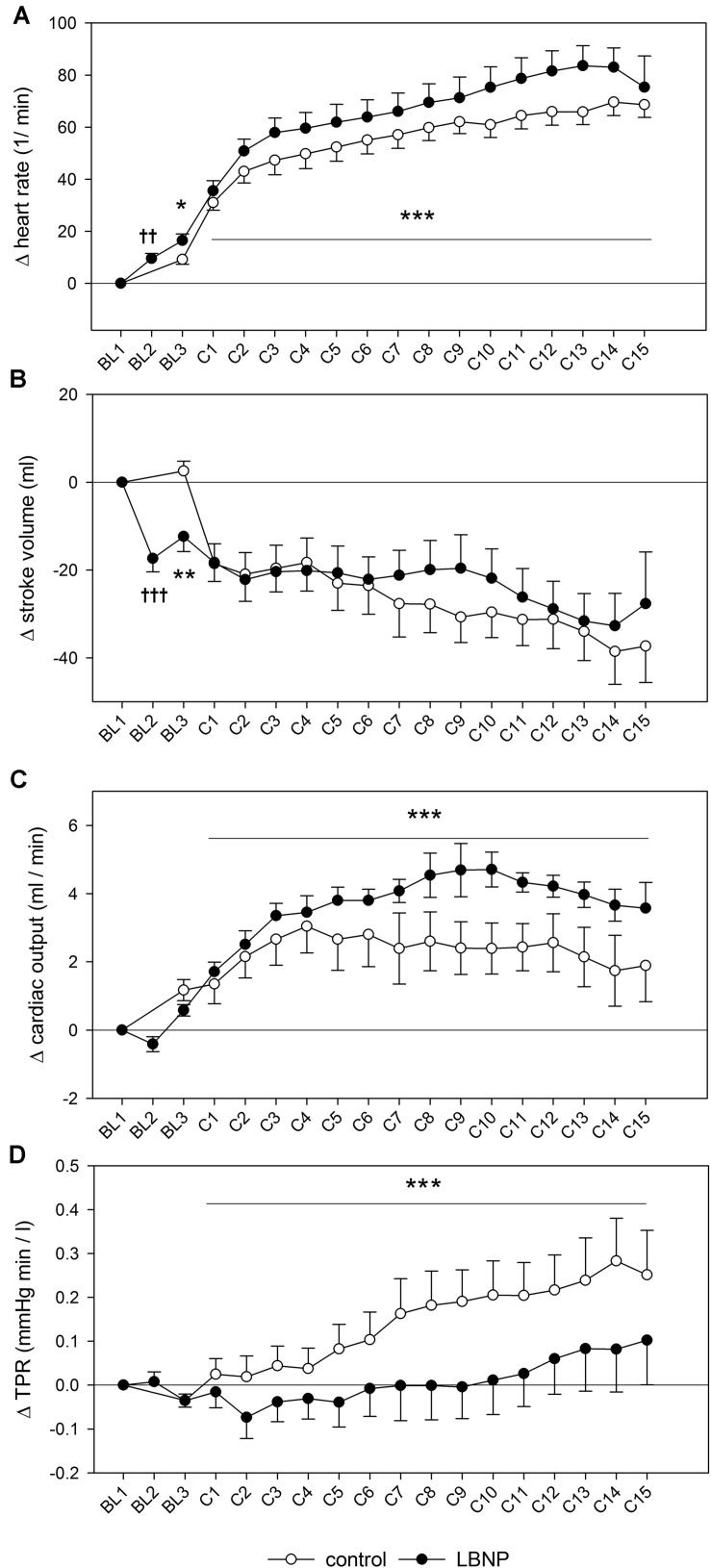
Heart rate **(A)**, stroke volume **(B)**, cardiac output **(C)** and total peripheral resistance (TPR, **D**) measured and depicted analogously to the blood pressure changes presented in Figure 3. Significant effects are marked as follows: † LBNP vs. baseline, and * LBNP vs. control, with increasing number of symbols corresponding to the level of significance: *p* < 0.05 (one symbol), *p* < 0.01 (two symbols) and *p* < 0.001 (three symbols).

Introducing LBNP caused a slight reduction in mean blood pressure during baseline data collection (BL2) by approximately 5 mmHg compared to the resting value (*d* = 2.3, *p* < 0.05), accompanied by a tendency toward lower systolic blood pressure (Figure 3). No change in diastolic blood pressure was found during baseline. Initiating LBNP caused an increase in heart rate compared to resting values by approximately 10 beats/min (*d* = 3.5, *p* < 0.01) and a concomitant reduction of SV by approximately 20 ml (*d* = 4.1, *p* < 0.001). Warm-up under LBNP further resulted in a more elevated heart rate compared to control (Figure 4). At baseline preceding the main exercise set (BL3), heart rate was elevated by approximately 20 beats/min under LBNP versus 10 beats/min under the control condition relative to the corresponding resting values (*d* = 1.2, *p* < 0.05). Concomitantly, there was a reduction in stroke volume by around 15 ml under LBNP, whereas its values remained stable under ambient pressure. Hence, baseline stroke volume was significantly reduced under LBNP (*d* = 1.8, *p* < 0.01). Cardiac output and total peripheral resistance (TPR) were, however, not affected by LBNP during BL2 or BL3.

Systolic blood pressure was higher by approximately 10 mmHg during the main exercise set under LBNP vs. control (*F* (1/153.2) 6.7, *p* < 0.05), without any accompanying changes in the mean or diastolic blood pressure (Figure 3). Increase in heart rate following the resting phase after warm-up (BL3) was also higher by approximately 10 heart beats under LBNP compared to control (*F* (1/202.2) 23.6, *p* < 0.001, Figure 4). Stroke volume overall gradually reduced with increasing contraction numbers, displaying no significant difference between the two conditions. The rise in cardiac output was approximately twice as high under LBNP versus the control condition (around 4 ml/min vs. 2 ml/min over the baseline value; *F* (1/176.9) 28.8, *p* < 0.001). TPR remained largely unchanged under LBNP and gradually increased by around 0.25 (mmHg x min)/l under ambient pressure. TPR thus became significantly higher under the control condition vs. LBNP (*F* (1/193.3) 31.9, *p* < 0.001).

### Muscle Hemoglobin Oxygenation and Respiratory Oxygen Uptake

**FIGURE 5:**
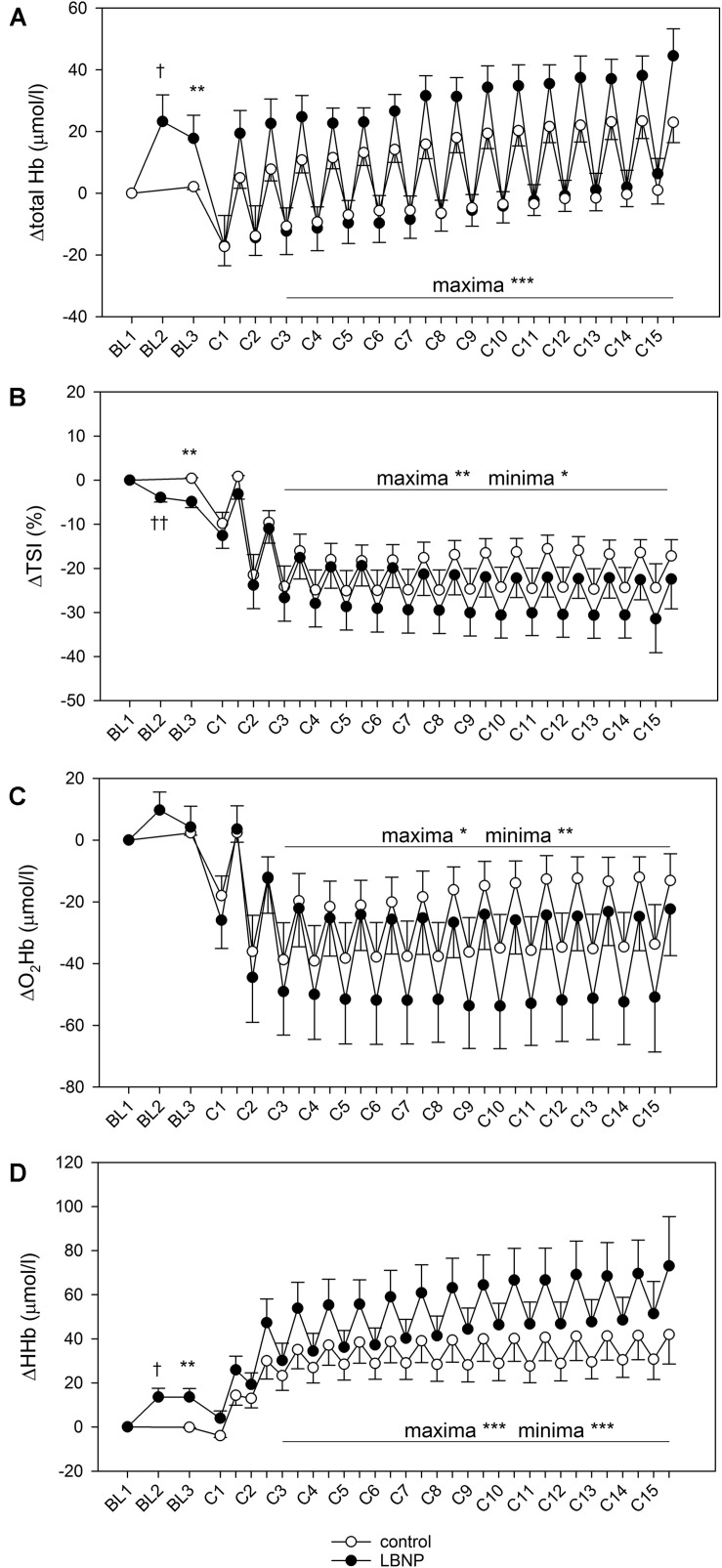
Total hemoglobin **(A)**, tissue oxygen saturation index (TSI, **B**), oxyhemoglobin (O_2_Hb, **C**) and deoxyhemoglobin content (HHb, **D**) of vastus lateralis during a single bout of resistive leg press exercise (contractions C1–C15) under ambient pressure (control, ∘; *n* = 9) or with LNBP (∙, *n* = 9), with subtracted onset baseline (BL1) values (Δ). Values are means ± SEM. BL2, LBNP onset; BL3, baseline directly preceding the main exercise set. Significant effects are marked as follows: † LBNP vs. baseline, * LBNP vs. control, with levels of significance analogous to Figure 4.

Activating the LBNP led to an increase in the total hemoglobin content (ΔtHb) of the vastus lateralis muscle by over 20 μmol/l compared to its initial values (*d* = 1.9, *p* < 0.05, Figure 5). Concomitantly, there was an increase in deoxyhemoglobin (ΔHHb; *d* = 2.4, *p* < 0.05) and a reduction of the tissue oxygen saturation index (ΔTSI; *d* = 2.7, *p* < 0.01), indicative of blood pooling in the lower limbs. Following the warm-up, ΔtHb was elevated by approximately 20 μmol/l under LBNP, whereas its level remained almost stable under ambient pressure, leading to a significant difference between the two conditions (*d* = 0.96, *p* < 0.01). This increase under LBNP was caused by elevated ΔHHb (*d* = 1.7, *p* < 0.01), while the oxyhemoglobin content (ΔO_2_Hb) remained stable. In consequence, a small difference in ΔTSI was found, its value being lower by around 5% with LBNP as compared to control (*d* = 1.8, *p* < 0.01).

Corresponding with alternating forces during the 15 contractions of the main exercise set, we observed a pattern of minima and maxima regarding the muscle hemoglobin content, as described in our previous findings (Parganlija et al., 2019). During the high force periods of the contractions, blood was pumped out of the muscle leading to depletion detectable as minimum hemoglobin values. The subsequent replenishment of blood supply during the low force periods lead to a refill detectable as maximum hemoglobin values, a pattern recurring with each contraction of the exercise set. For ΔtHb, minimum values were comparable between control and LBNP. However, its maximum values were consistently higher with LBNP by approximately 20 μmol/l vs. control (*F* (1/190.6) 50.0, *p* < 0.001), reflecting stronger blood refill under LBNP (Figure 5). ΔTSI was generally reduced during exercise, with a similar margin between the maxima and the minima in both groups of approximately 10%, indicating equivalent changes in the oxygenation status between the high force periods and the low force periods of the contractions. Over the course of the main exercise set, the ΔTSI values remained largely stable in the control group with maxima approximately 15% lower and the minima around 25% lower than baseline TSI. ΔTSI of the LBNP group initially displayed similar values, reducing however with increasing contraction numbers. Its values at the final contraction became approximately 35% for the minima and 25% for the maxima. Therefore, the overall ΔTSI in the main exercise set was significantly lower under LBNP compared to control (*F* (1/199.4) 6.5, *p* < 0.05 for minima and *F* (1/199.1) 7.2, *p* < 0.01 for maxima). The reduction in ΔO_2_Hb was more pronounced under LBNP, with both the maxima and the minima being significantly lower than in the control group (*F* (1/201.0) 4.7, *p* < 0.05 for maxima and *F* (1/202.5) 9.3, *p* < 0.01 for minima). This is attributed to rising values with increasing contraction numbers in the control group, a tendency that did not occur under LBNP. Notably, the margin between the ΔO_2_Hb minima and maxima was consistently higher under LBNP compared to control (around 30 μmol/l vs. 20 μmol/l, respectively). In contrast to ΔO_2_Hb, the ΔHHb levels were significantly elevated under LBNP (*F* (1/190.8) 17.8, *p* < 0.001 for minima; *F* (1/186.3) 24.8, *p* < 0.001 for maxima), due to higher initial values as well as a gradual increase during exercise not observed in the control group. In the LBNP group, the margin between the ΔHHb minima and maxima was approximately 20 μmol/l and therefore twice as high as in the control setting.

**FIGURE 6:**
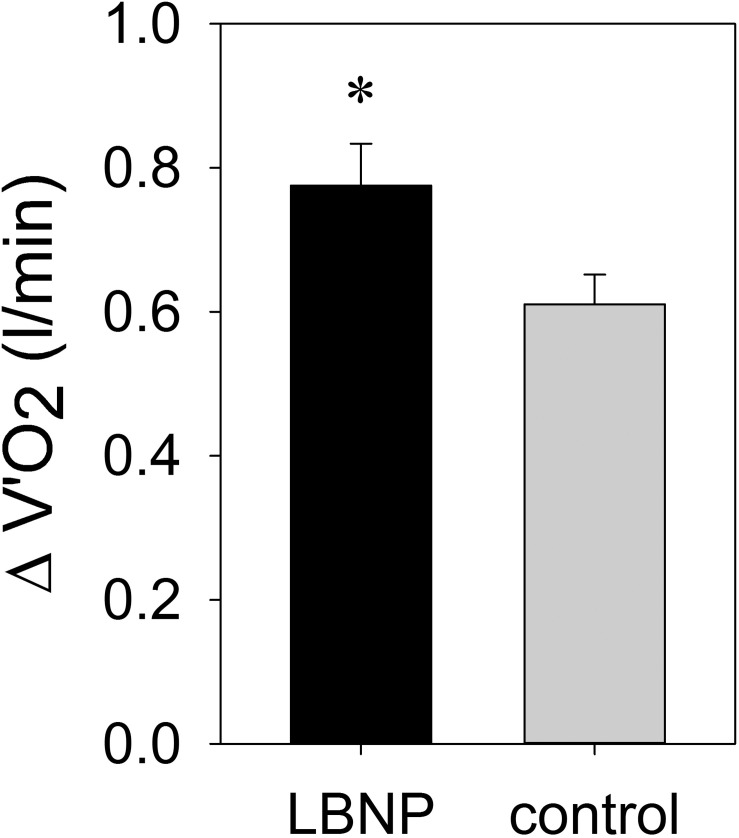
ΔV’O_2_ (mean ± SEM) was determined by spirometry during exercise under ambient pressure (control, gray bar; *n* = 9) or LBNP (black bar, *n* = 9). ^∗^*p* < 0.05, LBNP vs. control.

ΔV’O_2_ was significantly elevated under LBNP to 0.78 ± 0.06 l/min vs. 0.61 ± 0.04 l/min in the control group, indicating higher respiratory oxygen uptake compared to the control condition (*F* (1/16) 5.4, *p* < 0.05; Figure 6 and Supplementary Figure S1).

### Venous Lactate and Circulating Levels of Angiogenic Factors

**FIGURE 7:**
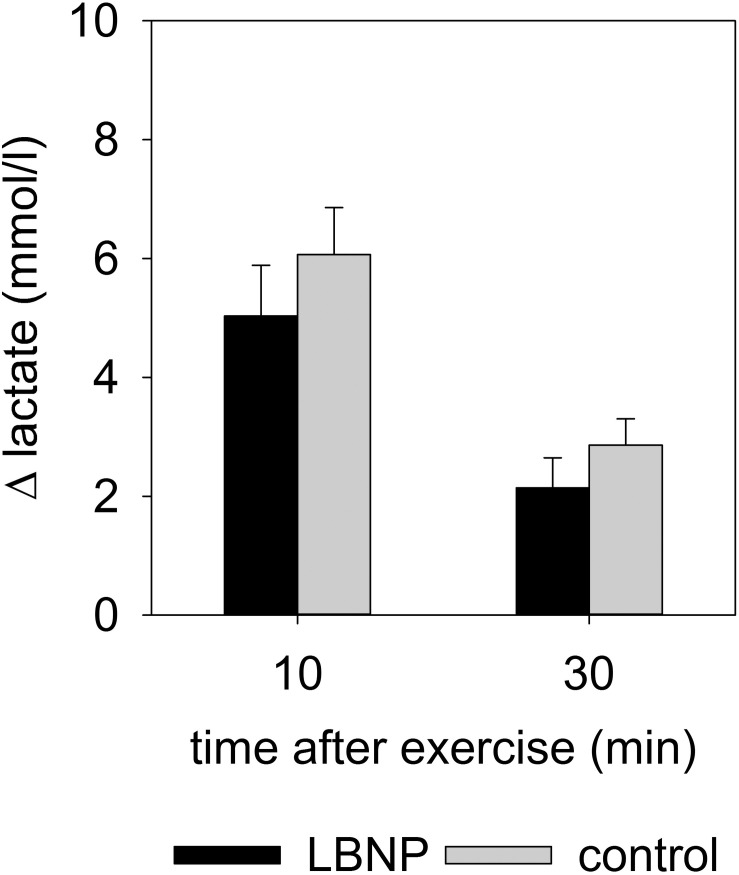
Δ lactate (mean ± SEM) in venous blood, 10 and 30 min after resistance exercise under ambient pressure (control, gray bars; *n* = 9) or LBNP (black bars, *n* = 9).

Comparable increase in venous lactate was found in both test groups, with the values 10 min post exercise at 5.0 ± 0.9 mmol/l (mean ± SEM) under LBNP and 6.1 ± 0.8 mmol/l in the control group, and 30 min post exercise at 2.1 ± 0.5 mmol/l under LBNP vs. 2.9 ± 0.4 mmol/l in the control group (Figure 7 and Supplementary Figure S2). Lactate levels in venous blood samples were therefore not significantly impacted by LBNP.

**FIGURE 8:**
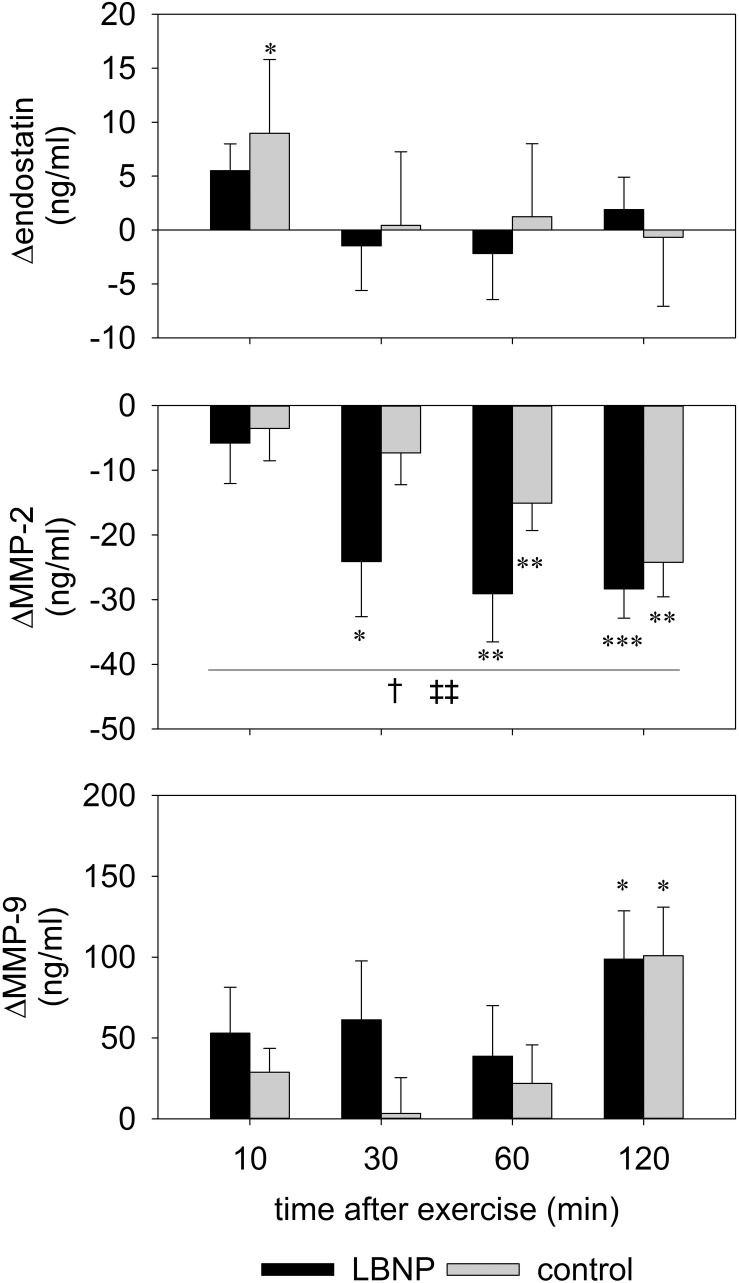
Serum levels of endostatin, MMP-2 and MMP-9 determined by ELISA of venous blood samples obtained prior to and 10, 30, 60, and 120 min post resistance exercise under ambient pressure (control, gray bars; *n* = 9) or LNBP (black bars, *n* = 9). Values are means ± SEM, with subtracted BL1 (Δ). Significant effects are marked as follows: ^∗^ sample (control or LBNP) vs. baseline, † LBNP vs. control, ‡ time effect, with levels of significance analogous to Figure 4.

Levels of circulating angiogenic factors endostatin, MMP-2 and MMP-9 were altered at various time points after exercise (Figure 8 and Supplementary Figure S3). An early reaction to the test protocol was observed with endostatin, with a small yet significant increase by around 9 ng/ml compared to baseline at 10 min after exercise under ambient pressure (average baseline value 102.2 ng/ml; *d* = 1.3, *p* < 0.05). Endostatin values of the control group subsequently recovered to baseline level at 30 min and remained stable up to the final measurement at 120 min after exercise. No significant change in the endostatin levels of the LBNP-group was found (baseline average 104 ng/ml). However, MMP-2 levels were significantly reduced in both groups compared to the respective baseline (control 230 ng/ml, LBNP 211.9 ng/ml). The reduction in MMP-2 occurred sooner under LBNP than in the control group (at 30 vs. 60 min post exercise, respectively). Moreover, the MMP-2 reduction was more pronounced under LBNP (*F* (1/60.4) 4.9, *p* < 0.05), reaching approximately 24 ng/ml as early as 30 min after exercise vs. 7 ng/ml in the control group. Whilst the MMP-2 levels of the LBNP-group remained relatively stable past the mentioned time point, the decrease occurred in a more protracted fashion in the control group, leading ultimately to somewhat similar levels at 120 min after exercise (decrease of around 28 ng/ml under LBNP, *d* = 2.9, *p* < 0.001, and 24 ng/ml in the control group compared to baseline, *d* = 2.1, *p* < 0.01). The MMP-9 response occurred later than the above described effects on endostatin and MMP-2, its levels not having been significantly altered in any of the two groups within the first hour after exercise. At 120 min after exercise, however, the MMP-9 levels of both groups were similarly elevated by approximately 100 ng/ml compared to respective baseline (average baseline values: control 189.6 ng/ml, LBNP 179 ng/ml; *d* = 1.6 and *p* < 0.05 for both conditions).

### AMPK-Phosphorylation in the Working Muscle

**FIGURE 9:**
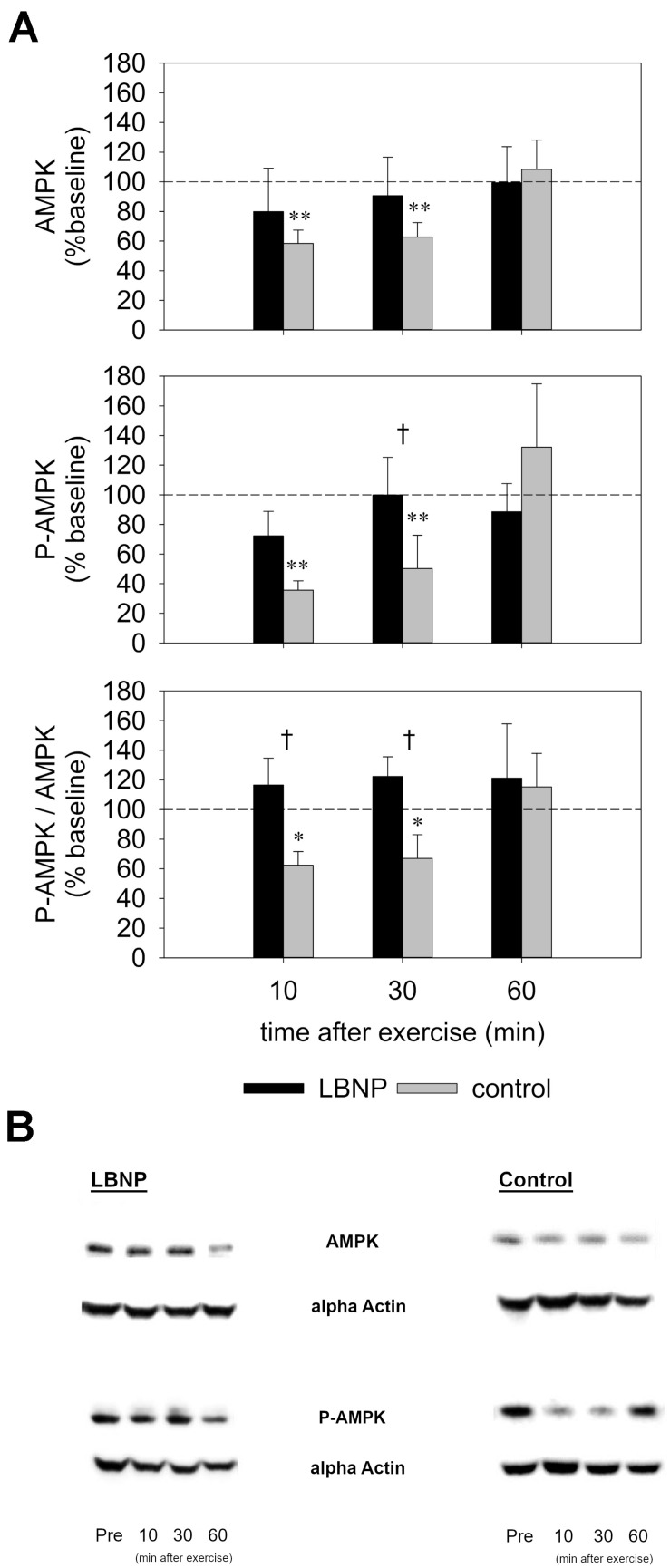
AMPK and phospho-AMPK levels **(A)** as determined by Western Blot of biopsies from the vastus lateralis (representative blots, **B**) obtained before and 10, 30, and 60 min after exercise under ambient pressure (control, gray bars; *n* = 9) or LBNP (black bars, *n* = 9). Results are displayed relative to baseline (%), values are means ± SEM. Significant effects are marked as follows: ^∗^ sample (control or LBNP) vs. baseline, † LBNP vs. control, with levels of significance analogous to Figure 4.

Subsequent to the main exercise set, the AMPK level was initially decreased in the control group by approximately 40% compared to baseline, as determined 10 min after exercise (*d* = 1.3, *p* < 0.01) and 30 min after exercise (*d* = 1.2, *p* < 0.01), and recovered to baseline values at 60 min following the exercise (Figure 9 and Supplementary Figure S4). No significant change in the AMPK level of the LBNP-group was found. In the control group, the P-AMPK level was also reduced by approximately 60% at 10 min (*d* = 1.4, *p* < 0.01), and around 50% at 30 min following the main exercise set (*d* = 1.2, *p* < 0.01), recovering to baseline values at 60 min after exercise. LBNP was not found to significantly impact the P-AMPK level respective to baseline at the followed-up time points. Nevertheless, the P-AMPK protein expression was significantly lower in the control group vs. LBNP at 30 min after exercise (*F* (1/16) 4.9, *p* < 0.05). In addition to the overall lower expression of the individual proteins, the P-AMPK/AMPK level of the control group was also reduced by approximately 40% at 10 min (*d* = 1.1, *p* < 0.05) as well as 30 min after exercise (*d* = 0.8, *p* < 0.05). Concomitantly, the ratio of the two proteins displayed a tendential increase under LBNP compared to baseline, which was not found to be statistically significant. However, a significant difference in the P-AMPK/AMPK ratio was determined between control and LBNP both at 10 and 30 min following exercise (*F* (1/16) 7.7 and *F* (1/16) 8.6, respectively; *p* < 0.05 for both conditions). As was the case with the individual proteins, the P-AMPK/AMPK ratio also recovered 60 min after exercise, establishing a return to pre-exercise protein expression.

## Discussion

In our present study, we compared the physiological and molecular responses to intense concentric-eccentric knee extension exercise in almost horizontal posture under ambient pressure as control versus under orthostasis simulated by LBNP. Exercise consisted of slow-paced contractions including low force periods allowing blood flow and high force periods representing an ischemic phase. The LBNP and control group were comparable in terms of 1-RM, age, body weight and height. As we have previously demonstrated, simulated orthostasis enhances the action of the muscle pump during supine exercise, with elevated capillary blood volume and oxygenation of the knee extensor muscles, and increased respiratory oxygen uptake suggestive of enhanced muscle perfusion and oxidative ATP formation (Parganlija et al., 2019). Beyond utilizing our previously established exercise protocol to explore the physiological effects of LBNP in a non-crossover study design, our present study aims to provide insights into the less investigated field of acute molecular responses to resistance exercise with LBNP. Our present research confirms some of our previous findings, in particular concerning enhanced muscle perfusion and oxygen uptake, and provides novel results in terms of the effects of LBNP on the response of the energy sensor AMPK in the muscle tissue and the circulating levels of angiogenic factors following the exercise stimulus.

Our current finding of a steeper EMG amplitude increase may indicate higher recruitment of large motor units over the course of the exercise with LBNP. However, all the measured parameters characterizing the exercise performance and the perceived exertion reported by the participants were comparable between the control and the LBNP group, suggesting equivalent work was accomplished in the two test conditions. Nevertheless, 3 out of 9 subjects of the LBNP group were unable to fully perform the main exercise set and terminated the exercise before completing their final repetition. Taken together, these observations suggest elevated fatigue under LBNP. This result stands in contrast to the one of our previous cross-over study that showed no significant difference in the EMG amplitude increase between the control and LBNP intervention (Parganlija et al., 2019). Our previous finding was accompanied by elevated oxyhemoglobin (O_2_Hb) levels in the vastus lateralis and higher oxygen uptake as well as higher lactate levels under LBNP, suggesting energy provision both from the aerobic and the anaerobic side. In the present study, we have also found indications of aerobic energy provision, but rather accompanied by a tendency toward reduced lactate levels. It is therefore possible that, as the exercise continued, rising energy demands could no longer be adequately met, resulting in metabolically induced fatigue and consequently a steeper EMG amplitude increase under LBNP. This conclusion is supported by elevated P-AMPK under LBNP, indicating higher AMP levels and therefore depleted energy in the exercising muscle. Previous research showed enhanced work performance and increased fatigue resistance under LBNP (Eiken, 1988; Zange et al., 2008). However, increased fatigue resistance was observed in a cross-over study applying ten bouts of 15 s maximum dynamic exercise in the form of load lifting intermitted by nine periods of 45 s rest (Zange et al., 2008). This setting of concentric muscle work differs strongly from our protocol involving high-load concentric and eccentric contractions. As electrical activation has previously been found to dominate in eccentric rather than comparably induced concentric contractions (Newham et al., 1983), this distinction in the exercise protocols might at least partly explain the differing findings.

In accordance with previous research and our own earlier findings, we have observed increased resting heart rate (HR) under LBNP (Eiken and Bjurstedt, 1985; Eiken et al., 1986; Nishiyasu et al., 1999; Parganlija et al., 2019). Elevated HR during exercise supports previous findings on bicycle and treadmill exercise with LBNP (Bonde-Petersen et al., 1984; Groppo et al., 2005) as well as our previous observation with intense resistive leg press exercise (Parganlija et al., 2019). However, it also contrasts the results of research on dynamic leg exercise in supine position that showed steady-state HR not to be significantly impacted by LBNP (Eiken and Bjurstedt, 1985; Eiken et al., 1986). This difference could be explained by HR not having reached a steady state in our study. Elevated HR contributed primarily to increased cardiac output (CO), as we observed no significant impact of LBNP on stroke volume (SV). Our finding of an elevated CO contrasts previous research that showed LBNP to reduce CO and also attenuate its exercise-induced increase in supine, incremental-load exercise on a cycle ergometer (Eiken and Bjurstedt, 1985; Eiken, 1987, 1988). However, decreased CO and SV have previously been discussed as signs of reduced venous return at rest as well as under exercise with LBNP (Bonde-Petersen et al., 1984; Eiken and Bjurstedt, 1985; Blaber et al., 2013). Therefore, our findings of elevated HR and CO as well as decreased total peripheral resistance (TPR) and elevated total hemoglobin (tHb) in the vastus lateralis muscle altogether suggest increased blood supply to the working muscle and support our notion of higher oxygen provision under LBNP.

Our finding of elevated tHb is consistent with previous research on LBNP both at rest (Nishiyasu et al., 1999; Hachiya et al., 2004; Bartels et al., 2011) and during exercise (Zange et al., 2008). However, contrary to the results of our earlier study (Parganlija et al., 2019), higher tHb was now accompanied by lower maxima of O_2_Hb and gradually declining maxima of the tissue oxygen saturation index (TSI). Our current finding also stands in contrast with previous research that showed LBNP not to influence tHb or hemoglobin oxygenation in thigh muscles during supine exercise on a cycle ergometer (Nishiyasu et al., 1999). On the other hand, LBNP has also been found to increase both tHb and O_2_Hb during dynamic load lifting in supine position (Zange et al., 2008). LBNP effects therefore apparently vary depending on the accompanying exercise stimulus. Interestingly, we currently observed a notably higher margin between the minima and maxima of O_2_Hb under LBNP than under ambient pressure. This observation suggests our relatively short, but intense bout of concentric and eccentric exercise possibly led to greater exploitation of the supplied oxygen under LBNP. This notion is further supported by the gradually rising levels of deoxyhemoglobin (HHb) and the accompanying decline of TSI under LBNP, suggesting a temporal effect on the oxygen exploitation with gradual stimulation of oxidative metabolism over the course of the exercise.

The observed higher oxygen uptake and a tendency toward lower lactate levels coupled with altered hemoglobin levels in the muscle tissue suggest oxidative metabolism might be providing a higher contribution toward energy production under LBNP. In light of elevated HR, CO and systolic blood pressure, it is, however, plausible that increased cardiac work rate might also contribute to the higher oxygen uptake. In any case, increased oxygen uptake under LBNP is consistent with previously described increased oxygen consumption (Groppo et al., 2005) as well as the findings of our own prior study (Parganlija et al., 2019). Furthermore, a benefit of LBNP in maintaining peak oxygen consumption during bed rest when combined with supine treadmill exercise has also been previously reported (Watenpaugh et al., 2000; Lee et al., 2007, 2009). Increase in blood lactate levels induced by exercise on a cycle ergometer was found to be attenuated by LBNP (Eiken, 1987), which is somewhat consistent with our results, as we have observed a trend toward lower lactate levels with LBNP. Facilitated lactate oxidation due to higher oxygen availability under LBNP may also have contributed to this finding. Lower blood lactate levels during cycle ergometer exercise with LBNP in supine position were accompanied by enhanced work performance attributable to a more efficient muscle blood flow resulting from increased local perfusion pressure (Eiken, 1988). These results correlate well with our observations of enhanced peripheral blood supply.

As gelatinases are released by different cell types into the bloodstream, their circulating levels are consequently dependent on the balance between release and resorption, and ultimately reflect a systemic response to exercise, including a contribution from the working muscle (Lo Presti et al., 2017). Higher levels of MMP-9 following exercise are consistent with previous studies on circulating levels of gelatinases that have generally shown an acute release of MMP-9 triggered by exercise of sufficient intensity (Lo Presti et al., 2017). Cycling has been shown to activate MMP-9 in the skeletal muscle without affecting endostatin or MMP-2 (Rullman et al., 2007). A slight increase in circulating MMP-2 and a marked increase of MMP-9 by almost half were found 10 min following a single bout of muscular endurance resistance exercise (Ross et al., 2014). MMP-9 activity increased in the vastus lateralis muscle after a single bout of supine dynamic constant-load knee-extension exercise in a similar fashion without and with blood flow restriction, with MMP-2 activity remaining unaffected (Rullman et al., 2009). These findings are partly supported by our results, as we have also found MMP-9 to increase in a comparable fashion in both our control and LBNP group. However, we have rather observed differential regulation of angiogenic factors in response to our exercise protocol, since increased levels of MMP-9 were accompanied by a reduction of MMP-2 levels in both groups. Our findings therefore support previous research that showed serum MMP-2 transiently decreases immediately following 60 min of cycling at submaximal intensity, and recovers only 2 h following exercise (Nourshahi et al., 2012). Release of gelatinases from storage within tissue as well as their transport within the bloodstream and therefore the measurable levels at the site of detection might be impacted by the local perfusion effects and cardiovascular response to LBNP. Nevertheless, a stronger decrease of MMP-2 under LBNP could suggest that the angiogenic stimulus is overall more suppressed after exercise with LBNP. Increase in endostatin directly following exercise under ambient pressure indicates early suppression of angiogenesis that did not occur in the LBNP group. This finding can at least partly be explained by decreased AMPK activation in the control group, as AMPK signaling cascade has been found to increase VEGF production in muscle and promote angiogenesis in response to ischemic injury (Ouchi et al., 2005).

AMPK phosphorylation is known to decrease in the early stages of muscle unloading (Vilchinskaya et al., 2015, 2018). This response is also reflected in our results, as we have observed an initial decrease in AMPK, phosphorylated AMPK (P-AMPK) as well as the P-AMPK/AMPK ratio in the control group, with the levels recovering 1 h after exercise. Furthermore, our findings suggest that said impact of supine position as a simplified model of altered perfusion comparable to microgravity is not modified by intense resistance exercise. On the other hand, our exercise protocol with LBNP led to stable levels both of AMPK and P-AMPK, bringing forward the conclusion that this combination is able to ameliorate their otherwise occurring initial decrease. Cycling has previously been shown to activate AMPK, with progressive phosphorylation occurring as the exercise continued (Wojtaszewski et al., 2000, 2002; Stephens et al., 2002; Rose et al., 2005). In our study, the ratio of P-AMK to AMPK was only found to be elevated with LBNP 10 and 30 min after exercise, with the levels recovering 1 h following exercise. This suggests that a short bout of intense resistance exercise can lead to a rapid increase in AMPK activity, provided a sufficient blood supply to the working muscle. In light of the higher EMG amplitude under LBNP, it is, however, also conceivable that elevated AMPK and P-AMPK compared to the control group might perhaps stem from a cumulative effect of increasing numbers of active motor units rather than elevated expression and phosphorylation in individual muscle fibers. Enhanced recruitment of motor units would in turn lead to higher oxygen exploitation, which is supported by the observed increase in tHb accompanied by a gradually declining TSI, in contrast to relatively stable TSI levels during exercise under ambient pressure. Transient hypoxia due to higher oxygen utilization of the working muscle and the subsequent absence of LBNP directly following exercise may also have initially contributed to the higher P-AMPK to AMPK ratio compared to the control group, as hypoxia is one of the known triggers of AMPK activation (Mu et al., 2001; Jørgensen et al., 2006). Elevated AMPK activation could contribute to initial maintaining of angiogenesis, which is supported by the lack of an early increase of endostatin such as the one observed in the control group.

In summary, we have found that combining LBNP with slow-paced, high load leg press exercise elicits a pattern of acute physiological responses indicative of enhanced peripheral blood supply to the working muscle and gradually enhancing oxygen exploitation in the muscle tissue. Our research further provides novel insights into the accompanying molecular responses to LBNP characterized by distinct changes in the circulating levels of angiogenic factors and enhanced post exercise AMPK activation in the muscle tissue. Our findings indicate superimposed LBNP modifies the impact of intense resistance exercise on the local metabolism of the working muscle and on its surrounding extracellular matrix. The observed pattern of molecular responses suggests a possible benefit for aerobic energy provision, as well as for muscle growth, since angiogenesis has been found to accompany hypertrophy (Holloway et al., 2018). LBNP provides useful means of studying the mechanisms underlying the adaptations to altered muscle perfusion and the orthostatic response. Continued research into the combination of LBNP and exercise provides a valuable contribution to the development of countermeasures for muscle loss in astronauts and possibly for future medical applications on Earth.

## Data Availability Statement

All datasets generated for this study are included in the article/Supplementary Material.

## Ethics Statement

The study involving human participants was reviewed and approved by the North Rhine Medical Association’s Ethics Committee. The participants provided their written informed consent to participate in this study.

## Author Contributions

DP, SG, JR, WB, and JZ concept and design of research. DP and FH performed the experiments. DP and JZ analyzed the data and prepared the figures. DP drafted the manuscript. All authors contributed to the article and approved the submitted version.

## Conflict of Interest

The authors declare that the research was conducted in the absence of any commercial or financial relationships that could be construed as a potential conflict of interest.

## Supplementary Material

FIGURE S1 Boxplot ΔV’O_2_.

FIGURE S2 Boxplot Δ lactate.

FIGURE S3 Boxplot angiogenic factors.

FIGURE S4 Boxplot (P-)AMPK.

## Abbreviations

1-RM: 1-repetition maximum
AMPK: AMP-activated protein kinase
BL1: baseline 1 (study phase)
BL2: baseline 2 (study phase)
BL3: baseline 3 (study phase)
BSA: bovine serum albumin
C: contraction
CO: cardiac output
ECG: electrocardiogram
EMG: electromyography
Hb: hemoglobin
HHb: deoxyhemoglobin
HR: heart rate
LBNP: lower body negative pressure
LME: linear mixed effects model
MMP: matrix metalloproteinase
NIRS: near infrared spectroscopy
O_2_Hb: oxyhemoglobin
P-AMPK: phospho-AMPK
RCL: robotically controlled leg press
RPE: rating of perceived exertion
rpm: revolutions per minute
SD: standard deviation
SDS-PAGE: sodium dodecyl sulfate polyacrylamide gel electrophoresis
SE: standard error
SEM: standard error of the mean
SV: stroke volume
tHb: total hemoglobin
TPR: total peripheral resistance
TSI: tissue oxygen saturation index
V’O_2_: respiratory oxygen uptake.
