# Supplementary figures and images for: Enhanced Blood Supply Through Lower Body Negative Pressure During Slow-Paced, High Load Leg Press Exercise Alters the Response of Muscle AMPK and Circulating Angiogenic Factors
Source: Front Physiol. 2020 Jul 30;11:781. doi: 10.3389/fphys.2020.00781 (PMC7406804; doi:10.3389/fphys.2020.00781)

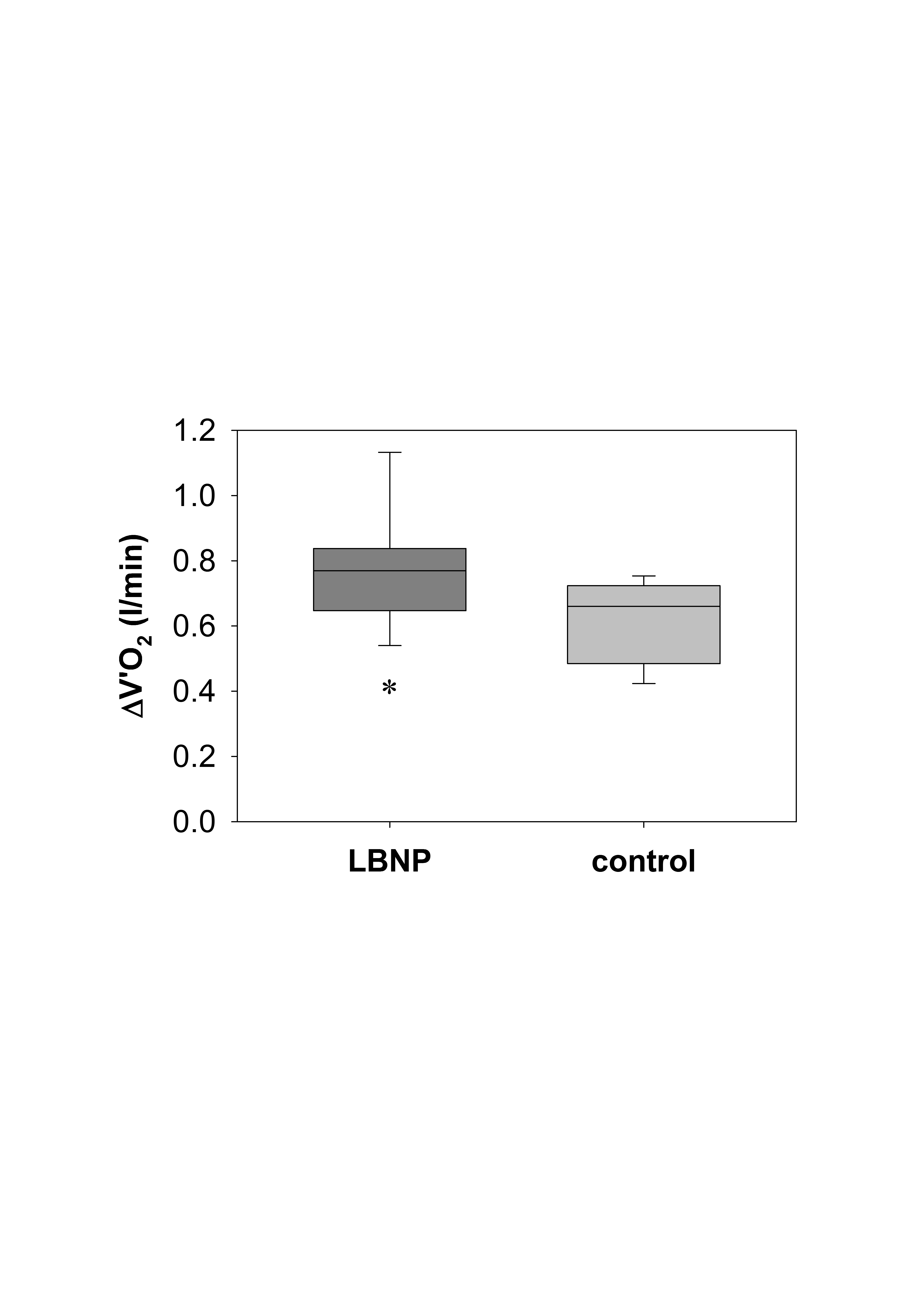

Supplement: FIGURE S1 — Boxplot ΔV’O2. [file Image_1.JPEG]

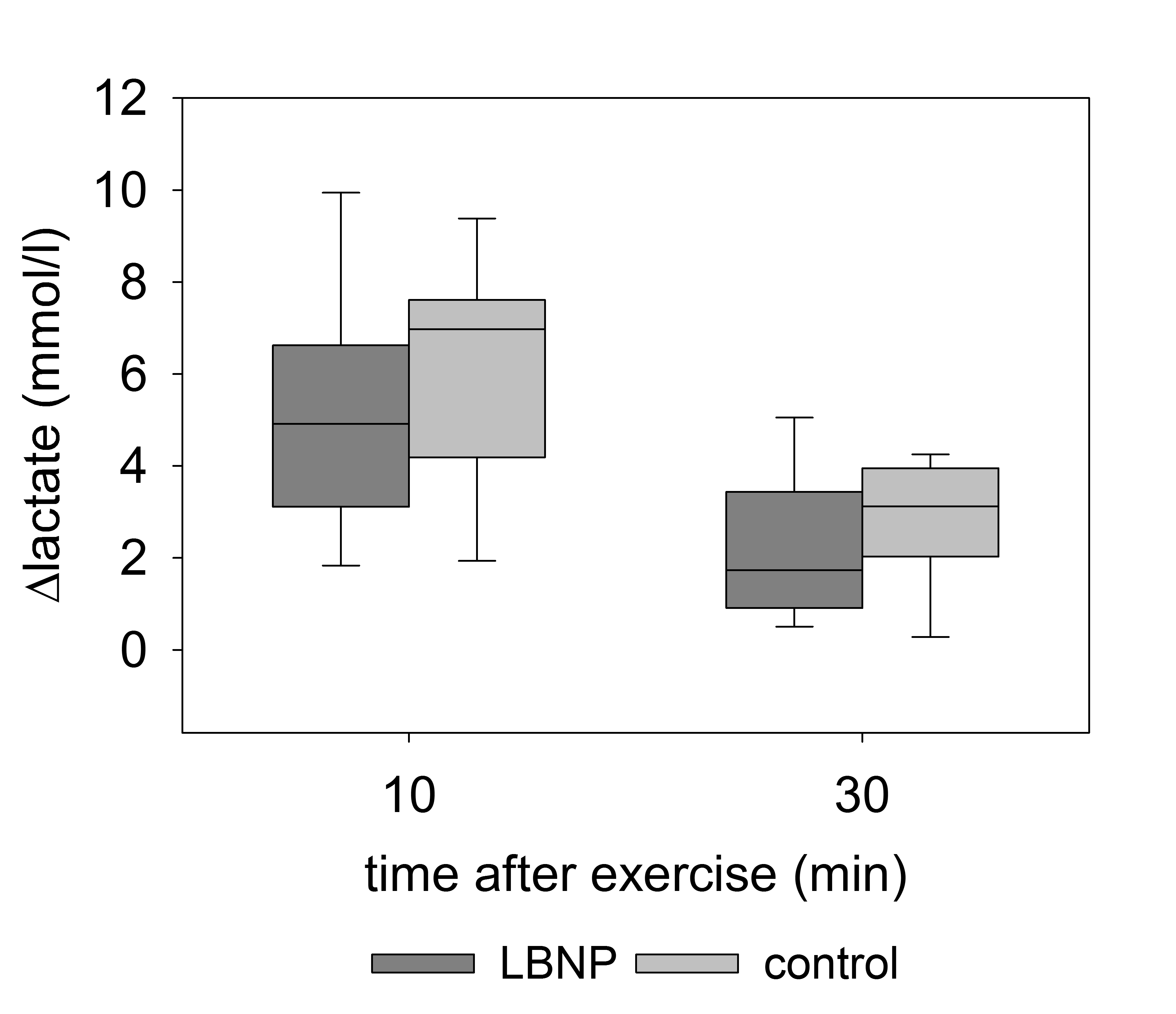

Supplement: FIGURE S2 — Boxplot Δ lactate. [file Image_2.JPEG]

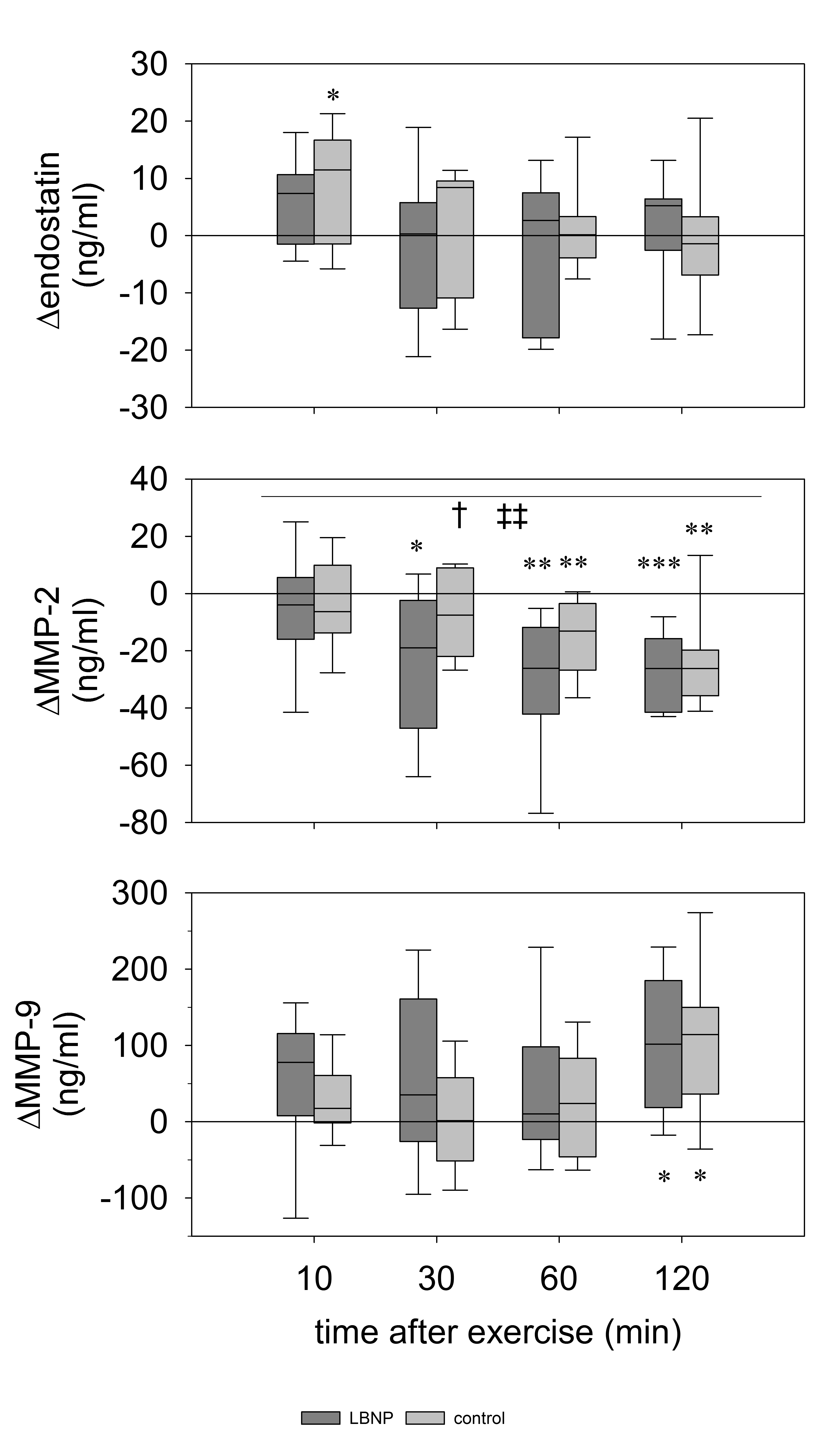

Supplement: FIGURE S3 — Boxplot angiogenic factors. [file Image_3.JPEG]

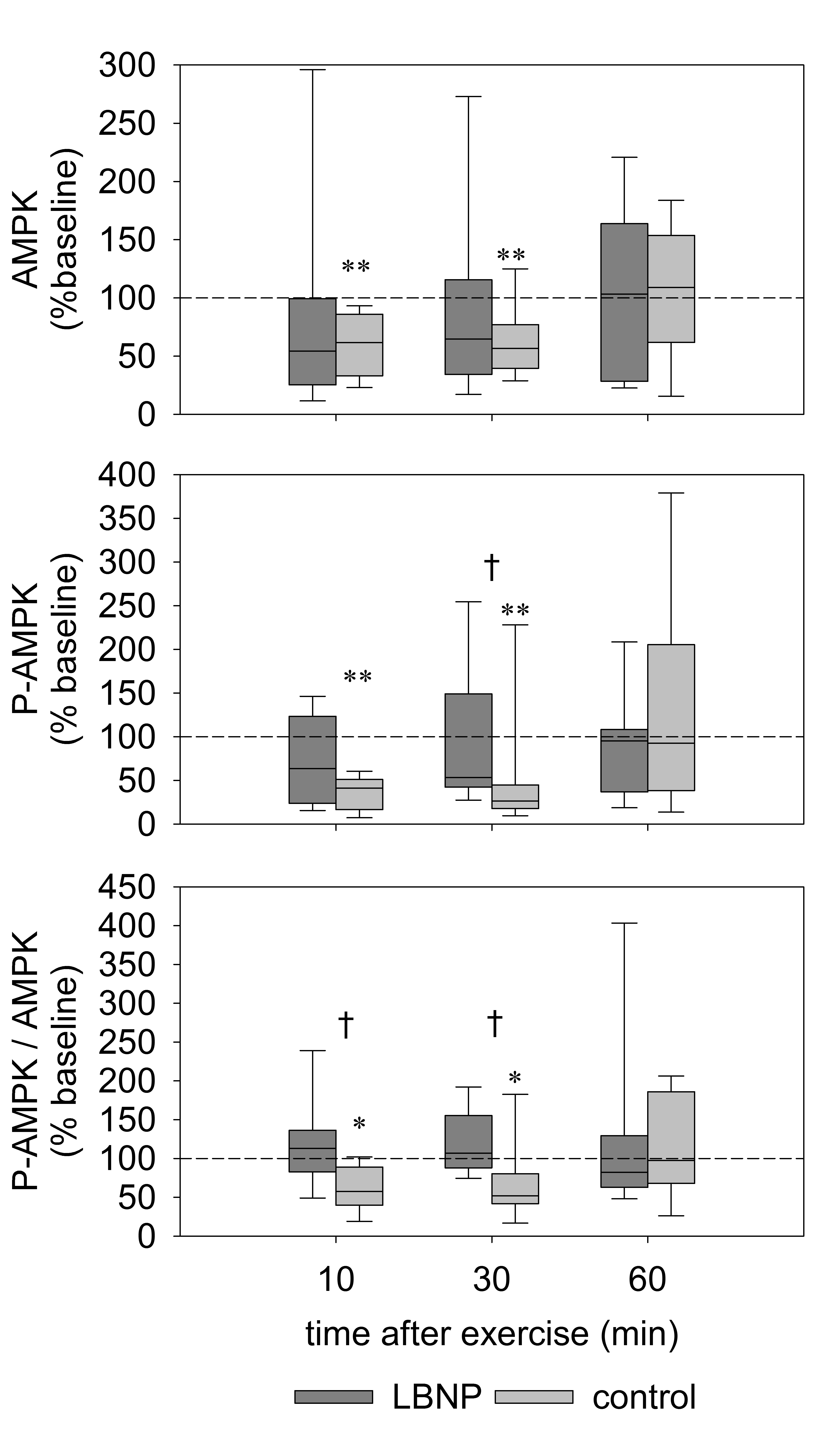

Supplement: FIGURE S4 — Boxplot (P-)AMPK. [file Image_4.JPEG]
